# Thermoplasmonic effect onto Toad physiology signals by plasmonic microchip structure

**DOI:** 10.1038/s41598-021-96640-w

**Published:** 2021-08-26

**Authors:** S. Akbari, S. M. Hamidi, H. Eftekhari, A. Soheilian

**Affiliations:** 1 Magneto-Plasmonic Lab, Laser and Plasma Research Institute, Shahid Beheshti University, Tehran, Iran; 2 Plasma Physics Research Center, Science and Research Branch, Islamic Azad University, Tehran, Iran

**Keywords:** Optical techniques, Diagnosis

## Abstract

Cardiovascular diseases are considered as the leading cause of death and almost 80% of deaths from this disease are developed in poor and less developed countries where early detection facilities are less available, along with overlooking the importance of screening. In other words, real-time monitoring of the physiological signals using flexible and wearable biosensors plays an important role in human life style. Thus, the present study aims to propose two dimensional flexible and wearable gold covered plasmonic samples as a physiological signal recorder, in which chips with nano array of resonant nanowire patterns performing in an integrated platform of plasmonic devices. The produced surface plasmon waves in our main chip were paired with an electric wave from the heart pulse and it use for recording and detecting the heartbeat of a toad with high accuracy. This measurement was performed in normal state and under external laser heating process to check the ability of signal recording and also thermoplasmonic effect onto the toad's heart signal. Our results show that our sensor was enough sensitive for detection while raising the body temperature of the toad and changing its heart rate as flatting T and P waves by thermoplasmonic effect.

## Introduction

Monitoring and distinguishing human physiological signals by portable, flexible, and wearable substrates play an important role in designing and constructing healthcare sensing devices^1,2^. Regarding the main objective of designing and fabricating flexible and wearable health monitoring devices, there are a lot of reports onto flexible batteries^3^, stretchable display^4^, and head of flexible main sensors^5,6^. These head devices are listed as main categories such as flexible piezoresistors^7^, capacitors^8^, carbon nanotubes^9^, ZnO nanowires^10^, or plasmonic nanostructures^11^ which can integrate with clothes^12,13^, eye glasses^14^, wristwatches^15^, and even skin sensors^7,16^. Enhancing signal-to-noise ratio, fast response, and remote sensing importance have motivated scientists to design and fabricate all optical sensors based on plasmonic nanostructures^17,18^. These nanostructures which can sustain collective electron oscillations at their surface to control electromagnetic field localization have been widely used for fabricating the mentioned flexible sensors based on plasmonic enhanced resonators^19,20^, Raman scattering^20^, and photovoltaic^20^ onto different substrates.

Actually, in these periodic arrays, metallic nanoparticles are physically separated by a period comparable to the wavelength of the incident light which have the ability to scatter light to produce diffracted waves and then a constructive interference of the optical fields from individual scatters can lead to a stronger exciting phenomenon, named as plasmonic surface lattice resonance(SLR)^21^ on the effect of shape and size of the nanoparticles and also the refractive index of construction materials. This phenomenon is currently used in a wide range of applications in science and technology; such as biosensing, photovoltaics, photocatalysis, much less it’s promising relationship with for example, spectroscopic techniques, Solar cells, Magneto-Optics and specifically Liquid Crystals^22–25^, random lasing^26^, optical filters^27^, and photothermal treatment by thermoplasmonic effect^28^.

This efficient and periodic thermoplasmonic effect in two-dimensional (2D) plasmonic nanostructures can help us design and fabricate the head of remote sensor by the aid of 2D plasmonic structures onto flexible substrates to be used as a physiology signal recorder. Among more than seven types of flexible substrate, silk^29^ and polydimethylsiloxane (PDMS)^30^ are the most popular and biocompatible substrate to integrate nanosensor devices which must be prepared in the lowest thickness in a miniaturized sensor. To obtain low cost flexible sensor, the ability of another famous polyimide substrate as Kapton film is used which can maintain its excellent physical, electrical, and mechanical properties over a wide temperature range^31,32^.

Moreover, different medical treatments and enhanced body temperature in the vicinity of heart or pulse in some diseases like heart arrhythmia can be controlled by neural stimulation and modulation based on photo thermal effects in all of optical processes^33,34^. Thus, nano-heaters can be helpful for enhancing the photo thermal effect and must be traced in heart beats. The results indicated the photo thermal effect of gold nanorods on enhancing neural activities in cells and nerves during infrared neural stimulation^35–38^. Furthermore, infrared neural stimulation of the sciatic nerve is enhanced by carbon nanoparticles^39^. In addition, a two-dimensional structure based on gold nanorods can help us benefit from infrared neural stimulation for regulating membrane depolarization^40^.

Innovations in miniaturized sensor technologies have made it possible to record electric impulses from heart in the absence of conventional electrocardiography (ECG) machines. Many of such technologies are wearable and can record cardiac impulses for long periods of time, which can lead to an increase in the utility of this technique in out-of-hospital settings such as households, endurance training, sports training, and public places. Further. it is possible to immediately transmit the obtained waveforms for expert interpretation, along with the already available computerized reports^8^. These sensors should have flexible and wearable properties, and should be small enough in size, which can be used for monitoring rhythms and waveforms over weeks or months. Thus, as another main part of remote sensors, all optical miniaturized and low size branches must be applied in the system. For this purpose, we use the pickup head of conventional DVD-ROM^41^ including a semiconductor laser diode, a lens for focusing on the laser beam, and photodiodes for detecting the light reflected from the disc surface.

The above-mentioned head and detection part present a simple plasmonic, low cost, flexible, and handheld sensor for physiological signal sensing and trace of photo thermal effect onto the heart beat instead of common ECG systems.

## Materials and methods

As the main head of signal recorder, 2D plasmonic nano structure has been fabricated by nano imprint lithography onto the Kapton substrate as follows. First, a CCD was extracted and separated from a camera. Then, a layer of the Kapton tape was placed onto the CCD by applying pressure. In the next stage, the sample was placed on the heater at 75 °C for 30 min. The sample was maintained under pressure at the room temperature for one week to stabilize the two-dimensional pattern onto the Kapton tape. Then, the Kapton tape was carefully removed from the CCD after one week, and a 2D flexible structure with very low thickness was achieved after thin gold film deposition by sputtering method.

**Figure 1:**
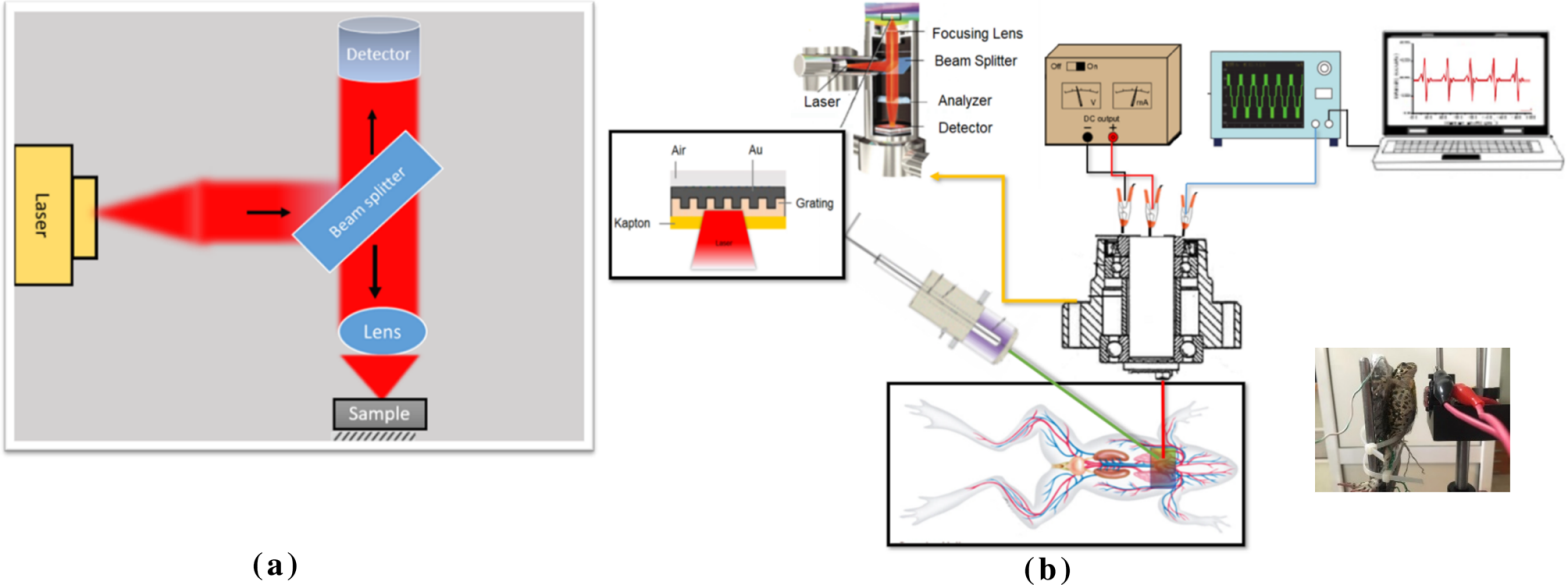
(**a**) optical setup of microchip and (**b**) main experimental setup which use for signal recording with and without green laser pumping and in the inset: real pic of experimental setup with toad.

After characterizing surface by scanning electron microscopy (SEM), atomic force microscopy (AFM) image and recording surface lattice resonance (SLR) of the sample in the visible region, microchip pickup head of conventional DVD-ROM was used (Fig. 1a). The head consisted of a semiconductor laser diode, 680 nm, with 5 mW power, a lens for focusing the laser beam, and photodiodes for detecting the light reflected from the 2D plasmonic surface which put onto the fixed toad, and three immature Eurasian green toads (weight 50–70 g) on the optical set-up (Fig. 1b). Finally, the reflected signals from toads were collected by digital oscilloscope.

As shown, the sample at SLR wavelength has more sensitivity and can distinguish and follow the input signal of the heart signal. There is the dependency of the reflections under p- and s-polarized incident light on the frequency and current from 2D plasmonic sample, which comes from the charge redistribution at the interface influencing the plasmonic resonance, especially SLR of the main head sample.

During the second step, continuous 532 nm laser as the pump ones fixed at the distance of 5 cm from the top of the toad to trace thermoplsmonic effect onto heart signals. First, the toad heart area was illuminated just by probe laser, and then plasmonic substrate was added to the heart area to determine the different effects of Au nano-rods heating and laser radiation. Regarding the required time for increasing the temperature, the heart area was irradiated for more than 5 min (Fig. 1b).

As shown, the microchip was placed on the top of the toad heart after fixing the green pump laser. The arrangement should be in such a way that the green laser can hit only the 2D sample on the toad heart without damaging the microchip. In this experiment, the temperature was measured by a thermocouple (TM-916, LUTRON electronic enterprise co, Taiwan). All of the tests were performed under the same conditions at a laboratory temperature of about 23 °C. After time and local heating, the microchip was turned on and the toad heart signal was measured after heating. According to this fact that use of anesthetics such as ether or chloroform interferes with the normal heart rhythm and heart rate of the toad^42^, no anesthetic was used in this experiment.

We can divide our experimental parts as follows: at first we record Toad's heart signal by the aid of probe red laser onto 2D plasmonic chip, in second step, we pump the heart by green pump laser without any plasmonic chip; thirdly, we use 2D plasmonic chip onto the heart and record signal by red probe beam when it pumps by green laser also.

When we shine a 532 nm pump laser at 10 Mw, a layer of plasmonic gold perforated 2D structure heats up on the sample, and transfers heat to the toad skin and the underlying tissue. To confirm the assumption, the sample was simulated in a commercial finite element method (FEM) solver, COMSOL Multiphysics 5.2.

To simulate surface lattice resonance (SLR) of the sample, Wave Optics Module and the Electromagnetic Waves, and Frequency Domain interface were used. In addition, a plane TE-polarized electromagnetic wave propagation was modeled in a single unit cell of a perforated gold layer on a Kapton substrate. Based on the electron microscope images, the thickness of the Kapton and gold layer were considered as 6 µm and 35 nm, respectively. The refractive index of Kapton is known as 1.89 and refractive index of gold is determined by Johnson and Christy’s dataset. This model was set up for one-unit cell, and its periodicity was described by Floquet boundary conditions.

### Sample guideline

All methods were carried out in accordance with relevant guidelines.

All experimental procedures were performed in accordance with guidelines and regulations approved by a regional Institutional Animal Care and Use Ethics Committee of the "Ethical committee of Vice president of research of Shahid Beheshti university/IR.SBU.REC.1405".

## Results and discussion

**Figure 2:**
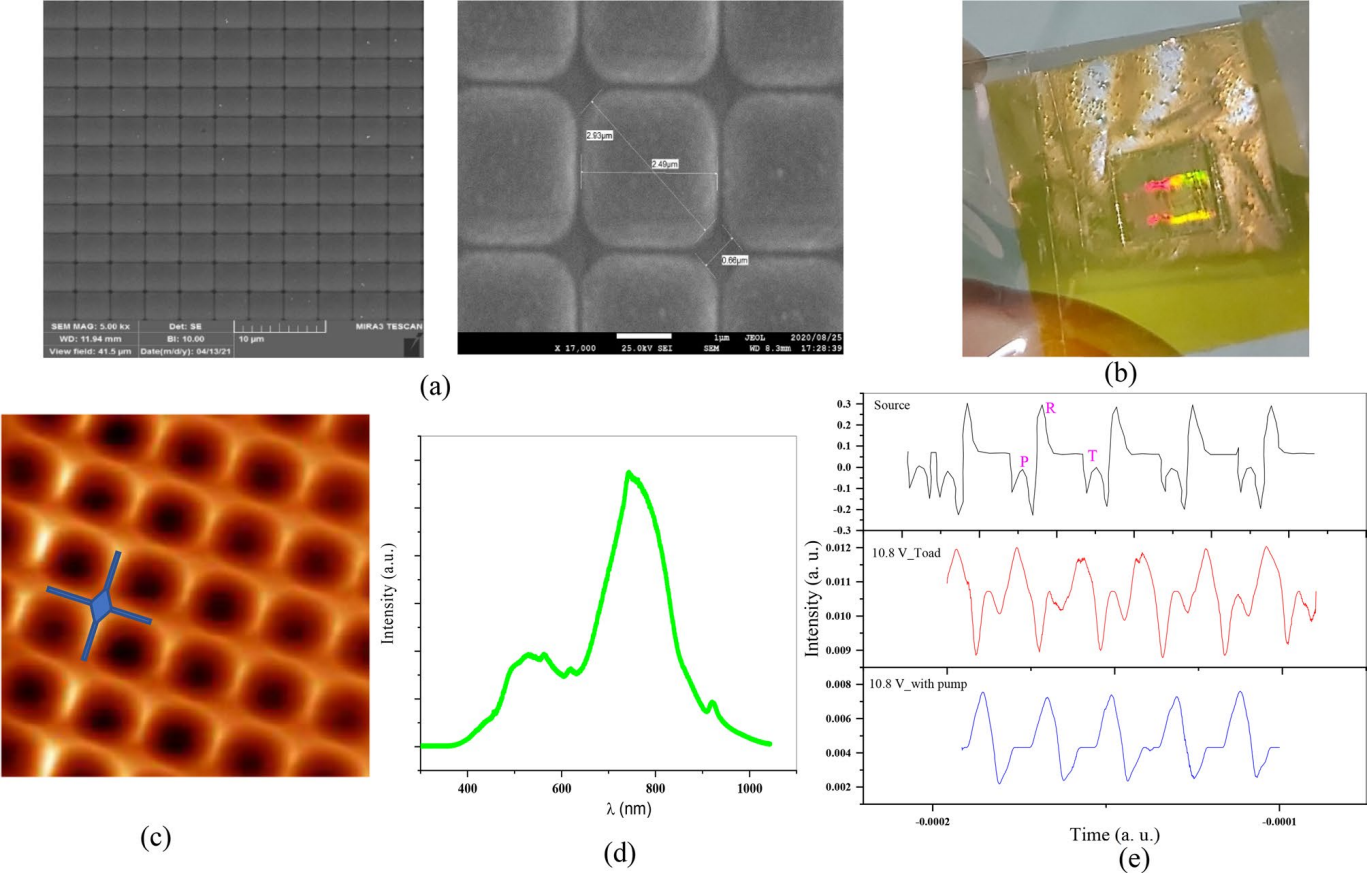
(**a**) SEM image of 2D plasmonic chip, (**b**) real picture of 2D flexible plasmonic chip onto Kapton and (**c**) AFM image of fabricated chip with wires region, (**d**) SLR response of the main 2D plasmonic chip, and (**e**) Heart signal recorded from toad as reference signal^49^, main signal and pumped ones.

Figure 2a displayed the SEM of the fabricated sample indicating nanowires in the corner of each unit cell by the size of 650 in 100 nm. In addition, real picture of the main flexible 2D plasmonic chip is shown in Fig. 2b. Gold wires production at each unit cell showed in Fig. 2 (c) by the aid of AFM image of the main 2D plasmonic chip. Due to lattice resonance of these wires, SLR is observed in the middle of visible region (Fig. 2d). Thus, we have good answer onto the detector from this sample in the visible region which must be useful as the sensor of flexible and ultra-sensitive physiological signal to heart pulse diagnostics. Due to the warming of the tissue, a sudden increase occurred in heart rate, and our sensor could detect this signal change well.

As explained above, heart pulses were recorded by the aid of our 2D plasmonic chip and microchip array, as shown in Fig. 2e. To get more sense about the heart signal, in the top row of this figure, we used the normal heart rhythm of this kind of toad picked up from^43,44^. In the middle row, the recorded pulses by 2D plasmonic chip were considered only by probe red laser. Pulses were recorded after green laser pumping onto 2D plasmonic chip and thus thermoplasmonic effect showed in the last ones (bottom row). Heart rhythm phases depend directly on the activity of the heart muscle and are affected by ionic changes within the heart cells. Due to the heating of gold Nanorods on a 2D plasmonic chip, the results of toad heart stimulation indicated significant changes in the activity of toad heart. Although no change was observed in heart rate when no thermal stimulation was performed and the toad was at normal temperature conditions, the effect of the green laser on the 2D sample and the heating of the gold nanorods had a significant effect on the heart rate. In addition, the ability of our sensor to detect and record the transmission of cardiac signals and their reproducibility is confirmed when the green laser was off.

In fact, when the plasmonic nanostructure is fixed on the toad heart and the immobile toad is placed at a suitable distance from the microchip, the toad heart signal recording is performed well. Thus, the signal recorded by our sensor matches very well with the heartbeat signal of an adult toad.

As shown in second row in Fig. 2e, no change in the amplitude of the heart rhythm phase was seen. However, the local temperature increase in the toad heart area due to the thermoplasmonic effect of gold nanorods onto the main 2D plasmonic grating, it affects the T and P waves amplitude. Compared to the toad heart rate rhythm before heat stimulation by green laser, the T and P-waves amplitude is significantly decreased.

The decrease and flattening effect in these waves amplitude is due to some biological effects on the toad's heart due to increase in temperature and heating.

It has been shown that increasing the temperature is effective in reducing the Action Potential time^45^ and thus decrease in T and P waves amplitude in our experiment onto toad's heart which is probably due to the increase in temperature that was transmitted to the underlying tissue through the toad's skin and affected the heart rate. Another reason is the increase in the concentration of calcium ions inside the cells^46^. It was shown that temperature increase by IR laser led to an increase in intracellular free Ca^2+^ concentration in ventricular cardio myocytes and cortical neurons^47,48^. In summary, our results show the photothermal effects that gold particles have on heart cells and thus on heart activity, and our sensor was able to accurately detect changes in the heart signal. In fact, our sensor had the ability to record the toad's heartbeat signal both in the normal state and in the state of change in heart activity.

**Figure 3:**
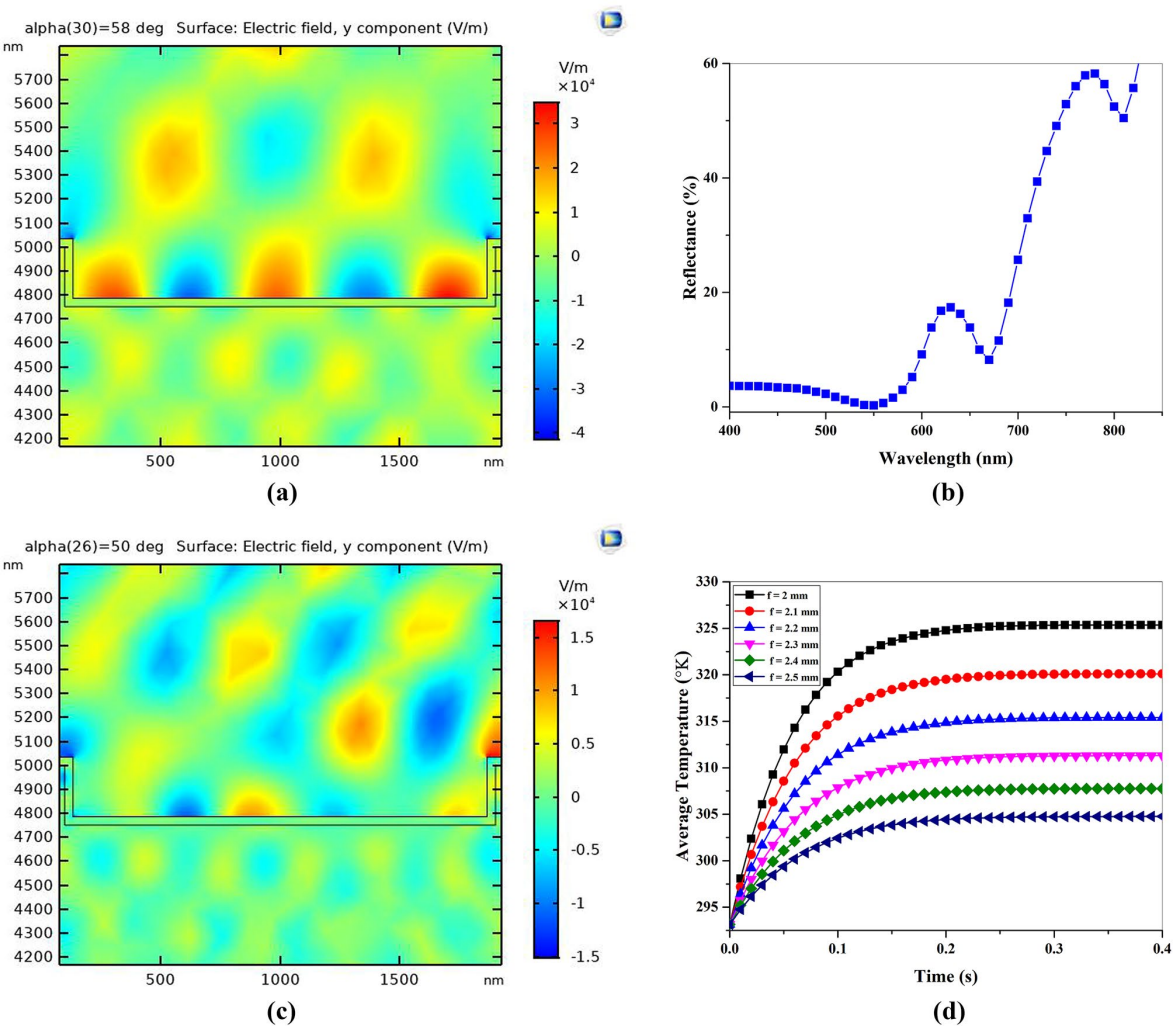
(**a**) Plasmonic SLR for propagation of plane wave at angle of 45° and wavelength of 680 nm, (**b**) transmittance, reflectance and absorption of the first order diffraction of plane waves for the single unit cell. (**c**) Plasmonic SLR for propagation of plane wave at angle of 45° and wavelength of 532 nm and (**d**) the average temperatures distribution across the sample for different distance of the laser focal point (h).

To get more sense about this thermoplasmonic effect, the main 2D plasmonic chip was simulated. In fact, the SLR of the sample was obtained as electric field distribution and reflectance spectra of the sample (Fig. 3a, b), which confirms that the plasmonic structure exhibits two resonant behaviors at 660 and 780 nm wavelengths by sweeping the wavelength.

It is well known that when nanostructures are arranged in arrays, the electromagnetic fields related to localized surface plasmon resonances of one nano wire (Fig. 3c) can affect the neighboring response. The narrow diffracted orders (DOs) and Localized surface plasmons resonance (LSPRs) in far-fields associating with individual nano rods couple together, SLR can takes place.

Then, the incident heat flux was modelled from the laser by green pumped laser, which distributed heat source spatially on the sample. First the electric field distribution was obtained at this wavelength as shown in Fig. 3c. Then, the transient thermal response of the sample was modeled with Heat Transfer in Solids Module in COMSOL. To this aim, the sample was irradiated by a Gaussian laser beam and heated for 0.4 min while the laser power was held constant at 10 mW. Figure 3d displays the probe plots of the average temperature distribution across the sample for different distances of the laser focal point (h).

**Figure 4:**
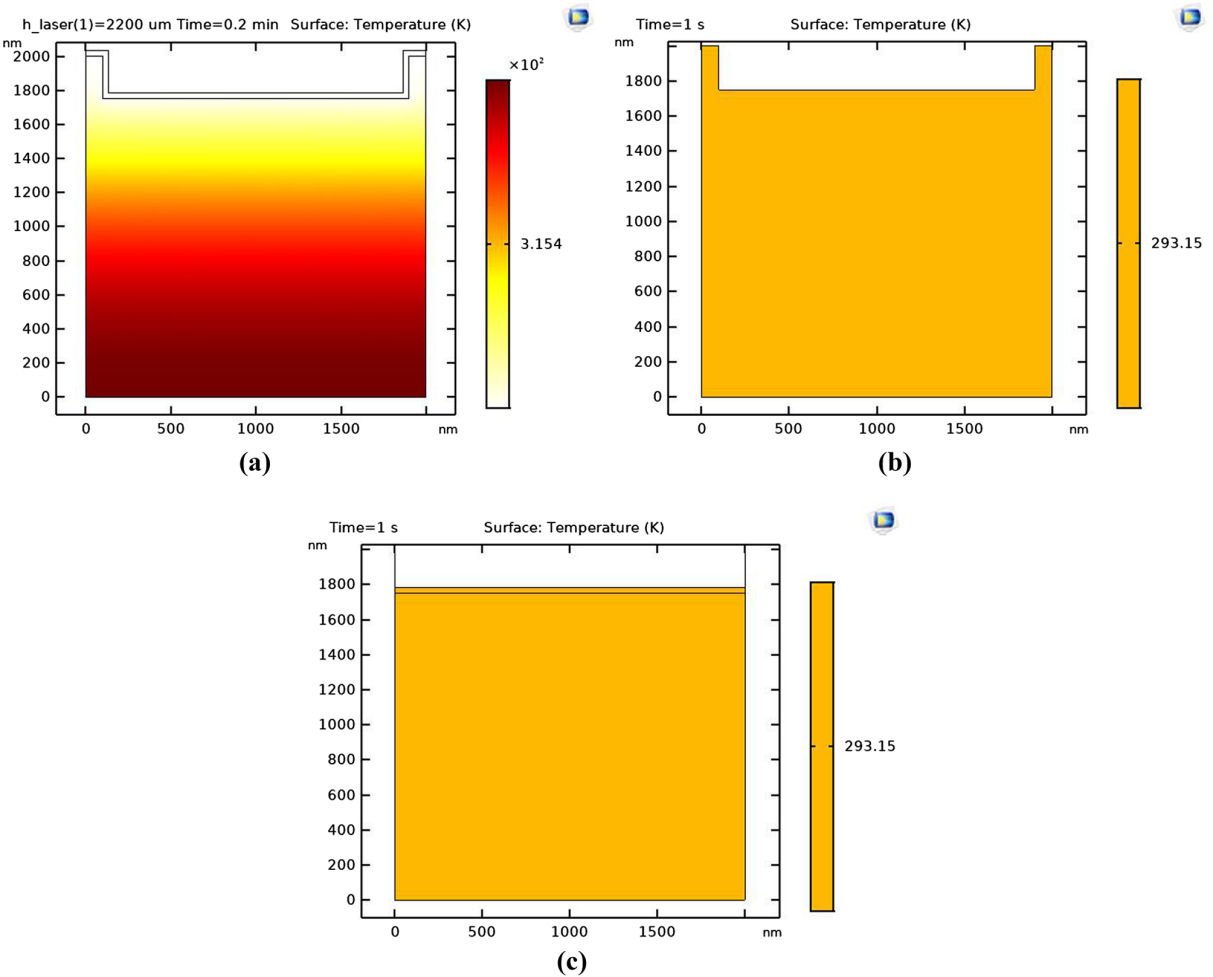
The temperature distribution across the (**a**) plasmonic sample with a nanostructured Au on Kapton substrate, (**b**) Kapton perforated substrate before gold coating and (**c**) Au thin film without any pattern.

As a control of experimental data, we simulated heat transfer of the nanostructure without gold thin film layer in addition of the main 2D sample. Figure 4 shows the temperature distribution plot across the main plasmonic sample, the Kapon sample without gold cover layer and thin gold layer to confirm the photothermal effect based on the 2D plasmonic nanostructure in the heart stimulation process.

The result shows no thermoplasmonic effect in the Kapton without gold thin film layer (As shown in Fig. 4b and also for thin non patterned gold thin films (Fig. 4c) which is approve this assumption that the periodic thermoplasmonic effect is created in 2D plasmonic nanostructures.

## Conclusions

In this study, new generation of wearable, small, flexible, and light heart rate recorders was proposed based on two dimensional plasmonic nanostructures as the main chip onto Kapton substrate. To reach the portable recorder, miniaturized optical setup was used near the heart surface of the toad. Based on the results, very good agreement was observed between input and output signal of heart beats in the toad and ability to distinguish between different signals. Finally, thermoplasmonic effect due to gold nanorods onto our main chip under green laser pump could affect the T and P shape of the heart signal, which was confirmed by the aid of the simulation part.
